# Potential for Therapeutic Alteration of the Underlying Biology of Epilepsy

**DOI:** 10.3390/biomedicines13092258

**Published:** 2025-09-13

**Authors:** Michael R. Sperling, Jurriaan M. Peters, Qian Wu, Michelle Guignet, H. Steve White, Evelyn K. Shih, Leock Y. Ngo, Enrique Carrazana, Adrian L. Rabinowicz

**Affiliations:** 1 Department of Neurology, Thomas Jefferson University, Philadelphia, PA 19107, USA; 2 Boston Children’s Hospital, Harvard Medical School, Boston, MA 02115, USA; 3 Center for Epilepsy Drug Discovery, Department of Pharmacy, University of Washington, Seattle, WA 98195, USA; 4 Independent Researcher, 461 From Road, Paramus, NJ 07652, USA; 5 Neurelis, Inc., San Diego, CA 92121, USA; 6 John A. Burns School of Medicine, University of Hawaii, Honolulu, HI 96813, USA; 7 Center for Molecular Biology and Biotechnology, Charles E. Schmidt College of Science, Florida Atlantic University, Jupiter, FL 33458, USA

**Keywords:** epilepsy, disease modification, anticonvulsants, antiseizure medication, immediate-use seizure medication, rescue medication

## Abstract

Approximately 30–35% of people with epilepsy experience seizures despite taking antiseizure medications. Recurrent seizures that are independent of status epilepticus can be associated with neuronal injury and structural changes to the brain, as well as diminished cognitive function, mood, and quality of life. A treatment that alters the underlying biology of epilepsy, thereby reducing the seizure burden and its attendant consequences, would be of great value in preventing these detrimental effects. In this review, we summarize preclinical and clinical research on pharmacological treatments that may favorably alter the underlying biology of epilepsy (i.e., disease modification or antiepileptogenesis). A reduction in seizures over time (e.g., increase in responder rates) or prevention of epilepsy in susceptible individuals has been observed with therapies that target neurotransmission (cenobamate, cannabidiol, vigabatrin, and diazepam nasal spray) and inflammation (everolimus), though evidence is limited and in preliminary stages. Pharmacological treatments that target neuroinflammation and oxidative stress have the potential to modify seizure phenotype and 1 or more comorbidities in preclinical studies (e.g., stress/anxiety and depression). Gene therapies and stem-cell-derived treatments also hold promise in reducing seizure burden in preclinical models, with several therapeutic candidates having advanced to phase 1/2 and 3 clinical trials. Effective disease-modifying strategies in epilepsy might include seizure control with novel antiseizure medications in combination with therapeutic targeting of key pathophysiological mechanisms. Standard criteria and a definition of disease modification should be established. Importantly, given the heterogeneity of the epilepsies, syndrome- or seizure-specific methods and trial design would likely be required.

## 1. Introduction

Epilepsy is a common, heterogeneous neurological disorder affecting approximately 50 million people worldwide [1,2]. Some epilepsies and their associated symptoms are progressive, whereas others may remain static or even improve over time [3,4]. The most common epilepsy treatments consist of chronic, daily antiseizure medications (ASMs) and intermittent rescue therapies (immediate-use seizure medications [ISMs]) for acute, as-needed treatment of seizure emergencies such as seizure clusters. As 30–35% of people with epilepsy continue to experience seizures despite the use of appropriate ASMs (i.e., drug-resistant epilepsy [DRE]) [5,6], other modalities such as neuromodulation and surgical therapy are also used [7].

Glutamatergic neurotransmission primarily mediates the increase in neuronal excitability in most epileptic seizures, along with a concurrent decrease in γ-aminobutyric (GABA)-ergic-mediated inhibitory neurotransmission [8]. Recurrent or continuous (i.e., status epilepticus [SE]) seizure activity can further alter the expression and localization of glutamate and GABA receptors as well as modify ionic gradients (e.g., chloride), reducing the seizure threshold and affecting pharmacoresponsiveness [9,10]. Glutamatergic-mediated increases in calcium signaling as well as production of reactive oxygen species may lead to neuronal injury and death [11]. On a larger scale, neural networks comprising functionally connected cortical and subcortical structures underlie epileptic seizures [12]. Secondary networks, where initiation, amplification, and expression of seizures occur at different sites, may explain how patients with Lennox–Gastaut syndrome can have similar electroclinical features despite differing etiologies [13].

Pharmacological treatments that produce long-term, persistent effects on disease course have been characterized in multiple neurological disorders, such as multiple sclerosis (MS), amyotrophic lateral sclerosis, Alzheimer’s disease, Parkinson’s disease, and migraine headaches [14,15,16,17,18,19,20]. For example, in MS, interferon β-1b, a modulator of the Janus kinase–signal transducer and activator of transcription (JAK-STAT) pathway, has been used for approximately 30 years in the US and EU [19,21,22,23]. Subsequent disease-modifying therapies have been introduced since then that use monoclonal antibodies to other targets, treating both relapses and disease progression [24,25].

Similarly, it may be possible to modify the disease course by altering the underlying biology of epilepsy. This may prevent worsening of seizure frequency, severity, or phenotype; improve pharmacosensitivity; or reduce the need for ASMs [26]. Secondary comorbidities (e.g., declining memory, depression, and anxiety) might also be improved and considered as a positive effect on the disease course [26,27]. A related concept, antiepileptogenesis, refers to the prevention of epilepsy in a person who has a risk factor for developing epilepsy, such as traumatic brain injury (TBI), brain tumor, infection, surgery, or stroke. In addition to targeting underlying physiological mechanisms to alter the disease biology, improving seizure control with medical management might alter the underlying biology of epilepsy. Currently, there are no ASMs available with a proven disease modification or antiepileptogenesis benefit in humans.

In this article, we discuss the negative effects of recurrent seizures and examine the potential long-term, persistent effects of pharmacological treatments on the course of epilepsy. Additionally, we discuss the possibility of altering the biology and natural history of epilepsy through the treatment of seizures and by modifying the underlying pathophysiological processes that lead to epilepsy.

## 2. Potential Harmful Effects of Recurrent Seizures on the Underlying Biology of Epilepsy

Recurrent seizures may produce a variety of negative effects, including neuronal loss and structural changes, for example, hippocampal and temporal sclerosis, cortical dysplasia and thinning, brain atrophy, and gliosis [4,28]. Progressive impairment of cognition may also be associated with recurrent seizures [28]. In a longitudinal, case–control study, cortical thinning was detected in 77% of patients with temporal lobe epilepsy (TLE), independent of age, with the most pronounced changes occurring within 5 years of the onset of seizures [29]. In a study that examined the hippocampi in patients with TLE, which is often drug-resistant, hippocampal volumes were 14% to 18% smaller in patients with drug-resistant TLE compared with healthy controls; there were no differences between newly diagnosed patients, those with chronic TLE and well-controlled seizures, and healthy controls [30]. Moreover, the magnitude of the reduction in hippocampal volume was associated with total seizure number [30]. In a systematic review and meta-analysis of patients with drug-resistant TLE, marked ipsilateral hippocampal atrophy was correlated with epilepsy duration (*r* = −0.42; *p* < 0.0001) and seizure frequency (*r* = −0.35; *p* < 0.0001) [31]. In a single-center study of patients with drug-resistant TLE, neuronal loss or dysfunction was associated with frequent generalized tonic–clonic seizures but not complex partial seizures (focal impaired consciousness seizures) [32], suggesting that seizure type influences neuronal or hippocampal volume loss. Lesions or atrophy of various brain structures have also been characterized in progressive myoclonic disorders [33] and SE [34]. In a prospective study of patients with a history of moderate to severe TBI and nonconvulsive electrographic seizures, hippocampal atrophy was greater in patients with TBI and seizures than in patients with TBI but no seizures (21% vs. 12%; *p* = 0.017) [35]. Whether this represents a causal effect is not known, as volume loss may be correlated with seizure propensity.

A review of cognition in patients with epilepsy identified 19 controlled studies, a subset of which investigated the association of seizure frequency with cognitive endpoints [36]. Associations were noted between seizure frequency and adverse effects on memory, executive function, and intellectual function in adults; improved seizure control was associated with a gain in IQ. While data are limited, there are reported associations between seizure frequency or poorly controlled seizures and adverse effects on intelligence [36]. In a recent study of children aged 36 months with tuberous sclerosis complex (TSC), cumulative seizure burden was the most outstanding predictor of neurodevelopmental outcome [37]. A cross-sectional study in adults found a learning peak at age 16 to 17 years in patients with TLE compared with a peak at age 23 to 24 years in healthy controls. A decline in performance then ran in parallel, and people with TLE had persistently worse learning. This underscores the importance of epilepsy control at an early age to prevent development impairment and injury [38]. Additionally, other factors/characteristics (e.g., medication and seizure type) in people with epilepsy have been associated with cognitive deficits [36]. Uncontrolled seizures, particularly tonic–clonic seizures, are associated with increased mortality rates from all causes and from sudden unexpected death in epilepsy (SUDEP) [39,40]. Frequency of tonic–clonic seizures relates to SUDEP risk, with a ceiling effect at 3 or more seizures per year [41]. Uncontrolled seizures are also associated with increased medical morbidity, risk of injury, hospitalization, and emergency department visits [42,43,44]. Epilepsy, regardless of etiology, can negatively influence the quality of life, for which seizure control is a considerable factor [45,46,47]. Persistent seizures are associated with increased rates of suicidality and post-ictal psychosis [42]. Additionally, depression, stigma, and fear of recurrent seizures, as well as diminished social well-being (less social engagement and fewer relationships) and ASM-induced adverse reactions, can all contribute to decreased quality of life [45,48,49].

## 3. Evidence for Potential Therapeutic Alteration of the Underlying Biology of Epilepsy

### 3.1. Preclinical Evidence

Preclinical studies are often employed to identify and characterize molecular mechanisms that might underlie human disease and relate to clinical outcomes. Small molecules, such as traditional ASMs, are the predominant therapeutic modality in the drug development landscape of epilepsy; however, emerging approaches such as gene therapies for genetic epilepsies, cell therapies for acquired epilepsies, and brain stimulation may transform underlying disease processes. In the following section, antiepileptogenesis was defined as the prevention, delay, or reduction in severity of the onset of epilepsy (spontaneous recurring seizures [SRSs]). Disease-modifying was defined as a modification of the disease course with established SRSs. Evidence of disease modification includes any positive change in SRSs, behavior, and/or pathology. A change in any of these domains qualifies as disease modification.

Results summarized in Table 1 suggest that a number of modalities (i.e., small molecules, cell-based therapies, and gene therapies) have been found to modify the seizure phenotype, behavioral comorbidities, and pathophysiology in animal models of genetic epilepsy (e.g., absence, CLN2 disease, and Dravet syndrome) and acquired epilepsy induced by TBI, viral encephalitis, kindling, and SE. At a mechanistic level, therapies that decrease neuroinflammation, reduce oxidative stress, block DNA hypermethylation (e.g., adenosine sparing), increase neurotrophic potential, and reduce neuronal excitability, including therapies that modulate glutamatergic or GABAergic neurotransmission, may modify disease (Table 1). For example, cannabidiol [50] and lamotrigine [51] have been shown to have disease-modifying and/or antiepileptogenic effects in acquired epilepsy models, whereas carbamazepine [52], valproic acid [53], phenobarbital [54], and lacosamide [55,56] either had no effect or minor effects in the post–SE-induced models. In animal models of absence epilepsy, ethosuximide [57] and perampanel [58] have been reported to be disease-modifying or antiepileptogenic. Unfortunately, none of these agents have been demonstrated to modify or prevent epilepsy in humans. The fact that ASMs are largely ineffective as disease-modifying therapies in post-SE models (despite their ability to reduce neuronal excitability and attenuate symptomatic seizures in animals and patients with epilepsy) is consistent with clinical studies in which phenobarbital, phenytoin, carbamazepine, valproate, and magnesium failed to prevent the development of posttraumatic epilepsy [59]. It is possible that other agents could yield different results, and the study of disease modification after SE and other brain injuries should not be abandoned.

The approaches with the greatest promise as disease-modifying therapies in preclinical studies include those therapies that target neuroinflammation and oxidative stress. These insults contribute to the development of epilepsy (Table 1). Many anti-inflammatory and antioxidant therapies modify both seizure phenotype and one or more comorbidities, including stress/anxiety [77,79], depression [61,62,79], and cognitive decline [65,76,77] associated with brain insult in animal models. However, soticlestat, an inhibitor of cholesterol 24-hydroxylase that reduces inflammatory markers, failed to meet primary clinical endpoints for seizure reduction in Dravet syndrome and Lennox–Gastaut syndrome [102], despite favorable results in a mouse model of Dravet syndrome [103]. Studies of anti-inflammatory agents are still worthwhile, as this agent used differently or other agents may prove beneficial. Disease-modifying results have also been observed in animal models with therapies that reduce neuronal excitability by replacing inhibitory interneurons within the epileptic circuit using targeted implantation of neuronal stem cells [87,91,92,93] or neurotrophic approaches using adeno-associated virus (AAV) vector-driven enhancement of inhibitory neuronal function [63,64,99]. These therapies will be discussed in the next section, as they have entered human trials, with preclinical studies guiding clinical development.

Target-driven therapeutic discovery offers hope for identifying specific disease-modifying treatments that may go beyond seizure control. Preclinical models provide platforms to evaluate these mechanisms in a controlled and systematic manner and allow scientists to explore the processes underlying epileptogenesis, such as neuroinflammation, oxidative stress, and disruptions in neuronal-glial interactions. This targeted approach opens the door to personalized therapies tailored to the pathophysiology of individual patients, potentially offering opportunities for disease modification and improved long-term outcomes.

### 3.2. Clinical Evidence

#### 3.2.1. Nonpharmacological Therapies

Surgical resection and neurostimulation can alter the trajectory of epilepsy and its comorbidities for patients with epilepsy. Surgical resection can result in seizure freedom, but surgery is appropriate for only a subset of patients with refractory epilepsy, and approximately half of the patients continue to experience seizures postresection [104,105]. However, even when seizures persist, surgery can abolish tonic–clonic seizures in many patients [106], and surgery is associated with reduced mortality [107]. In children with TSC, surgery has been associated with neurodevelopmental improvement [108], providing strong evidence that seizure control improves developmental outcomes.

Long-term neurostimulation is also appropriate for a subset of patients with refractory epilepsy and includes responsive neurostimulation (RNS), deep brain stimulation (DBS), and vagus nerve stimulation (VNS). Neurostimulation appears to alter neural (seizure) networks over time, reduce the likelihood of seizures [109,110], yield extended periods of seizure freedom with progressively reduced frequency over time [111], and reduce mortality risk [112]. In three open-label extension (OLE) studies, the median reduction in seizure frequency in patients with RNS at 2, 5, and 9 years of observation were 53%, 66%, and 75%, respectively [113,114,115]. In patients with DBS who participated in a randomized controlled trial, the median reduction in seizure frequency at 2, 5, and 7 years of follow-up were 56%, 69%, and 75%, respectively [116,117,118]. In a meta-analysis of patients treated with VNS, mean seizure reduction at 1, 2, and 3 years was 32.9%, 44.4%, and 53.5%, respectively [119]. While surgical and some neurostimulation strategies reduce seizures over time, suggesting a change in the natural history of epilepsy, there remains an unmet need for noninvasive, less restrictive options for which a heterogeneous patient population would be eligible.

#### 3.2.2. Pharmacological Therapies

There is evidence to suggest that some ASMs may have long-term, persistent effects on the course of epilepsy (e.g., progressive reduction in seizure counts, increases in responder rates, or other measures of seizure control over time). Pharmacological treatments that might alter the underlying biology of epilepsy include ASMs (daily and rescue treatments) as well as other treatments. The ability of an ASM to alter the underlying biology of epilepsy might result from direct action on the pathophysiology of epilepsy, indirect action as a product of enhanced seizure control (i.e., prevent negative outcomes associated with recurrent seizures), or some combination of the two. We have included recent clinical trials of ASMs that demonstrated favorable long-term improvements in seizure control, as these treatments might alter the biology of epilepsy as a result of fewer and/or less severe seizures (Table 2).

##### Daily Treatments

(1) Cenobamate

**Figure 1:**
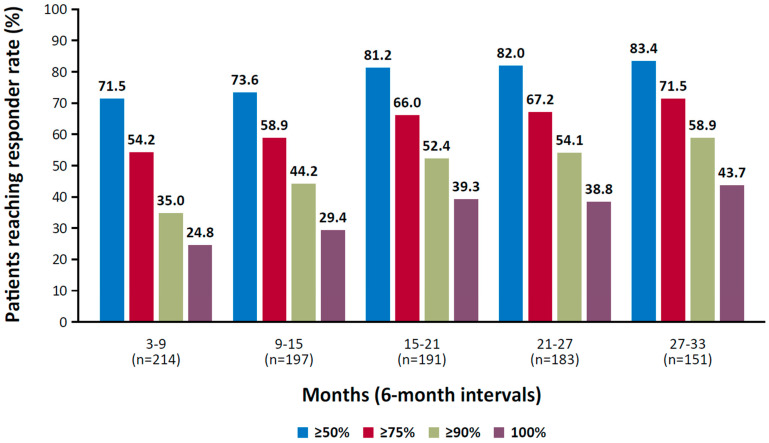
Responder rate at consecutive 6-month intervals in a phase 3, open-label study of cenobamate. Reproduced with permission from Sperling et al., Efficacy of cenobamate for uncontrolled focal seizures: Post hoc analysis of a Phase 3, multicenter, open-label study; published by Wiley, 2021.

Cenobamate, an inhibitor of voltage-gated sodium currents and a positive allosteric modulator of GABA-A receptors, has exhibited long-term efficacy in controlling seizures [120,132,133]. In a randomized, placebo-controlled trial of adult patients with focal seizures, the median percent change in seizure frequency during the 18-week treatment period was 55% in the 200 mg and 400 mg cenobamate groups compared with 24% in the placebo group (*p* < 0.001) [120]. In a long-term, open-label study, rates of participants who achieved seizure freedom during consecutive 6-month intervals increased over time, from 25% during months 3 to 9 to 44% during months 27 to 33 (median dose at month 33, 300 mg; Figure 1) [121].

(2) Cannabidiol

**Figure 2:**
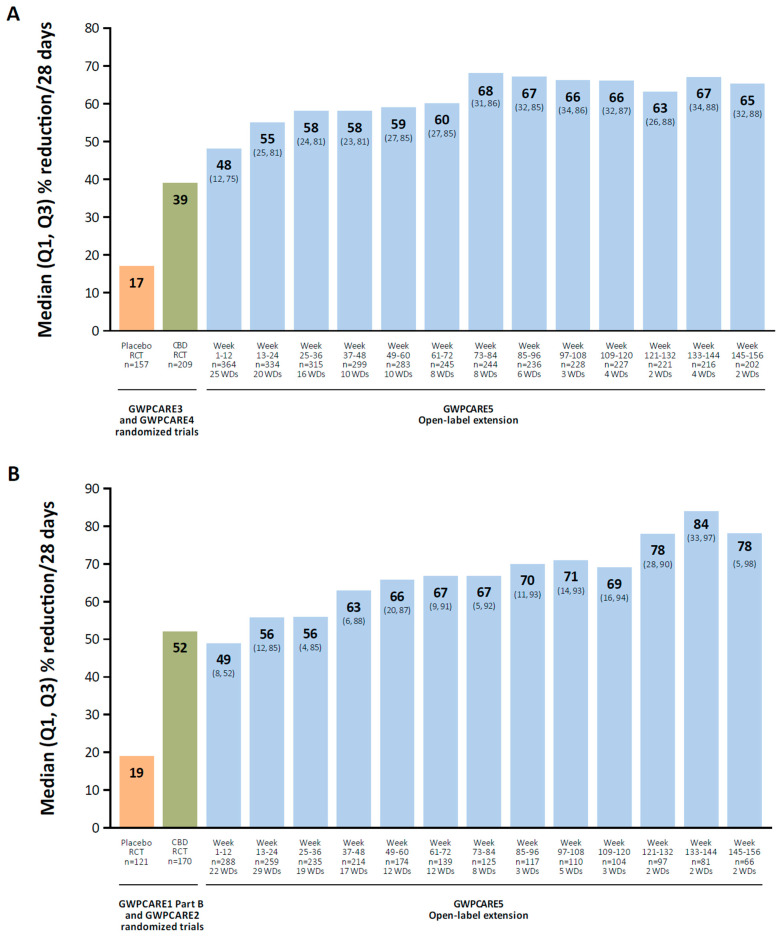
Median reduction in total seizure frequency in OLEs of cannabidiol oral solution. Patients with Lennox–Gastaut (**A**) or Dravet syndrome (**B**). CBD, cannabidiol; OLE, open-label extension; RCT, randomized controlled trial; WD, withdrawal. Reproduced with permission from Patel et al., Long-term safety and efficacy of add-on cannabidiol in patients with Lennox–Gastaut syndrome: Results of a long-term open-label extension trial; published by Wiley, 2021 and Scheffer et al., Add-on cannabidiol in patients with Dravet syndrome: Results of a long-term open-label extension trial; published by Wiley, 2021.

There is a wide variety of molecular targets for cannabidiol, including nonselective ion channels (transient-receptor potential channels), voltage-gated sodium channels, potassium channels, G protein–coupled receptor 55, and 5-HT receptors [134]. In a long-term OLE study of cannabidiol oral solution in patients with Lennox–Gastaut syndrome, the median percent reduction in total seizures increased over time, from 48% at weeks 1 to 12 to 65% at weeks 145 to 156 (mean modal dose, 24 mg/kg/d; Figure 2A) [122]. In a long-term OLE in patients with Dravet syndrome, the median percent reduction in total seizures increased from 49% at weeks 1 to 12 to 78% at weeks 145 to 156 (mean modal dose, 22 mg/kg/d; Figure 2B) [123].

(3) Vigabatrin

Vigabatrin is an inhibitor of GABA transaminase, a GABA-degrading enzyme; inhibition of this enzyme increases GABA availability and attenuates excitability associated with seizures [135]. It has been suggested that vigabatrin could alter the course or prevent the development of epilepsy in TSC [124,125,126]. In a retrospective study, 65% of children with TSC who received early treatment with vigabatrin achieved seizure freedom; in those who received vigabatrin later, 24% achieved seizure freedom (*p* < 0.01) [124]. In a prospective study of vigabatrin that compared early treatment (within a week of the appearance of epileptiform discharges in the EEG) with standard treatment (within a week of onset of seizures), infants with TSC and early treatment had lower rates of intellectual disability (14% vs. 48%; *p* = 0.031), fewer polytherapy requirements (21% vs. 55%; *p* = 0.039), and a lower incidence of DRE (7% vs. 42%; *p* = 0.004) [125]. This study was limited by its design—an open-label, single-center study that prospectively compared a preventative treatment group with a prior group of infants who had received standard treatment based on conventional practice [125].

In a pooled analysis from a randomized controlled trial (EPISTOP), children at 24 months of age who received prophylactic vigabatrin that was administered after epileptiform activity was detected but before onset of seizures had a lower likelihood of developing clinical seizures (OR, 0.21; 95% CI, 0.04–0.9), infantile spasms (OR, 0; 95% CI, 0–0.33), and DRE (OR, 0.23; 95% CI, 0.06–0.83) compared with children receiving conventional treatment (ASMs after the development of electrographic or clinical seizures) [126]. There were no differences in cognitive scores (Bayley Scales of Infant and Toddler Development, 3rd ed [Bayley-III]) between groups, and the incidence of neurodevelopmental delay was also similar [126]. Limitations of the trial were small sample size (only 27 children randomized) and the absence of a placebo-treated control group. In a larger randomized, double-blind, placebo-controlled trial (PREVeNT), no differences were observed between vigabatrin and placebo treatments on cognitive (Bayley-III) and behavioral (Vineland-II adaptive behavior) outcomes in infants with TSC, consistent with the EPISTOP study [126,127]. In both studies, infants in the comparator groups received open-label vigabatrin for seizure control once seizures developed. In the PREVeNT study, most infants in the placebo group experienced seizures soon after randomization, so the duration of vigabatrin exposure between placebo and vigabatrin groups was similar, potentially affecting the analysis of cognitive measures [127]. Moreover, there were no differences between vigabatrin and placebo in the number of infants who experienced seizures or had DRE at 24 months of age, which was not consistent with EPISTOP, although vigabatrin delayed the onset and reduced the prevalence of infantile spasms. Despite randomization, the TSC genotype was unbalanced between placebo and vigabatrin groups; infants with *TSC1* mutations or variants of unknown significance/no mutation genotypes were more common in the placebo arm. Therefore, the potentially milder form of TSC in the placebo group might have masked any between-treatment separation. Future trials should control for multiple TSC-associated factors that affect seizure risk, DRE, and cognitive outcomes [127].

##### Intermittent Treatments

Diazepam Nasal Spray

Diazepam is a positive allosteric modulator of the GABA-A receptor, and diazepam nasal spray is an aqueous spray that utilizes dodecyl maltoside (Intravail^®^) to facilitate intranasal absorption and vitamin E to enhance the solubility of diazepam [136,137]. The safety and effectiveness of diazepam nasal spray were examined in a phase 3, long-term, open-label, repeat-dose safety study conducted in patients with seizure clusters aged 6 to 65 years [138] and a phase 1/2a, open-label, single-dose, PK study with a 180-day, open-label safety period and optional extension enrolling patients with epilepsy aged 2 to 5 years [139]. Diazepam nasal spray had a comparable safety profile to diazepam rectal gel, and <20% of seizure clusters were treated with a second dose, supporting its effectiveness [138,139].

**Figure 3:**
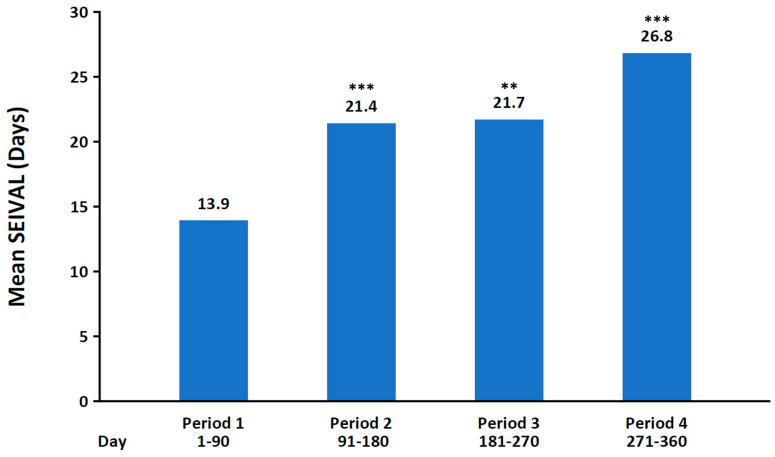
Mean interval between seizure clusters for Periods 1–4 of phase 3, open-label study of diazepam nasal spray (*n* = 76). SEIVAL, seizure interval. ** *p* < 0.01, *** *p* ≤ 0.001 compared with Period 1. Reproduced with permission from Misra et al., Significant improvements in seizure interval (time between seizure clusters) across time in patients treated with diazepam nasal spray as intermittent rescue therapy for seizure clusters; published by Wiley, 2022.

A post hoc analysis of the phase 3 study in patients aged 6 to 65 years was conducted to examine the duration (in days) between treated seizure clusters (seizure interval [SEIVAL]) over time [128]. SEIVAL was assessed using 90-day periods, as this period length would allow for the inclusion of a sufficient number of patients with an assessable SEIVAL for each period; four 90-day periods approximated the study’s 365-day treatment period. In the analysis of patients with SEIVALs in each of the four 90-day periods (second doses excluded [i.e., excluding retreatment of a seizure cluster within 24 h]), mean SEIVAL increased from 13.9 days in Period 1 to 26.8 days in Period 4 (*p* ≤ 0.001; Figure 3). The increase in SEIVAL was independent of age, duration of exposure, and change in concomitant medications. This was consistent with results for the overall study population, which included variable cohorts for each period [128]. Similar results were observed regardless of sex, whether the medication was self-administered or administered by a caregiver, or the presence of developmental epileptic encephalopathies [140,141].

A subsequent analysis of the open-label safety study evaluated the effect of treatment on the proportion of prolonged seizures recorded in the study population over time (Periods 1–4) [142]. In this analysis, prolonged seizures were defined as those treated 5 to 15 min after seizure onset. When anchored to the patient’s first treated seizure, the proportion of prolonged seizures decreased from 31% in Period 1 to 17% in Period 4. When anchoring the start of Period 1 to the patient’s first treated prolonged seizure, the proportion of prolonged seizures decreased from 44% in Period 1 to 15% in Period 4 [142].

The mechanism for the increase in SEIVAL should be elucidated. A preclinical, repeat-dosing method of diazepam has been developed in rats to create an experimental condition for intermittent use of diazepam similar to the rescue treatment of seizure clusters in patients [143]. If progressively increased SEIVAL is caused by rapid cessation of seizure activity or prevention of clusters, these preclinical studies will provide an opportunity to specifically evaluate the effects of intermittent diazepam administration on the underlying biology of seizures [143].

##### Other Pharmacological Treatments

Everolimus inhibits mammalian target of rapamycin (mTOR) signaling, which is involved in the pathophysiology of TSC [129]. In a randomized, placebo-controlled trial conducted in patients with focal onset seizures in TSC, the number of patients who achieved ≥50% reduction in seizure frequency (responders) from baseline was greater in low-dose (28.2%; *p* = 0.0077; 3–7 ng/mL) and high-dose (40.0%; *p* < 0.001; 9–15 ng/mL) everolimus groups compared with placebo (15.1%). Median percentage changes in seizure frequency from baseline were 14.9%, 29.3%, and 39.6% with placebo, low-dose, and high-dose everolimus, respectively [129]. In a separate study, the effect of everolimus on white matter in TSC was examined using diffusion tensor imaging [130], with changes in white matter having been associated with neurological comorbidity [144]. Everolimus treatment was associated with a decrease in mean diffusivity and radial diffusivity, while axial diffusivity increased in untreated patients, suggesting that everolimus modifies white matter changes that occur with increased mTOR activity in TSC [130]. Hence, treating underlying pathophysiology affects epilepsy outcomes.

A retrospective study conducted in pediatric patients with DRE who received pulsatile corticoid therapy (dexamethasone; up to 10 cycles) assessed whether treatment reduced the burden of interictal epileptiform activity [131]. Such burden was reduced after dexamethasone treatment compared with baseline; sleep physiology and quality of life (QOL) also improved following treatment. The authors concluded that pulsatile corticoid therapy may attenuate the pathophysiology of epilepsy and improve associated outcomes of sleep and cognitive function [131].

## 4. Discussion

Research is needed to develop therapies that produce long-term beneficial effects on the course of disease. The nature of epilepsy and epileptogenesis is variable and complex [145], and complex and innovative solutions will likely be required. Nevertheless, preliminary evidence suggests that pharmacological modification of the long-term disease course may be achievable. Agents that enhance inhibition, such as vigabatrin and diazepam, or address pathophysiological mechanisms associated with epilepsy, such as everolimus, hold promise for altering the natural history of epilepsy. Challenges that come with evaluating disease modification in clinical trials include establishing validated biomarkers for disease modification (which are likely syndrome-specific) that can demonstrate target engagement of the drug or intervention. Rigorous trial designs that use validated longitudinal endpoints, allow for variability of natural history, and have sufficient statistical power are necessary to identify successful disease-modifying therapies.

Focusing on the use of relevant animal models that display many parallels to the human condition should guide clinical trials. Data emerging from these studies can provide important insights into the pathophysiology of the disease and the potential for therapeutic interventions that target disease-specific defects. As described below, preclinical disease-modifying studies have the potential to inform clinical trials designed to demonstrate human disease modification and play an important role in providing proof of concept that the treatment, through engagement of its intended target, has the potential to modify the outcome in an appropriate animal model. Past experience suggests that animal models often best predict results in animal models, so no assumptions can be made about effects in human epilepsy without investigation in humans.

### Ongoing Research

Targeted therapies acting on underlying anatomic and physiological derangements have now entered clinical trials. Stem– or progenitor–cell-based interventions to replace and/or repair altered networks and gene/RNA therapies (e.g., AAV vectors and antisense oligonucleotides) are in the early stages of clinical development. In preliminary findings from an ongoing phase 1/2 clinical trial (NCT05135091) [146], adult participants with drug-resistant, unilateral TLE (*n* = 5) experienced 82% seizure reduction following transplantation of medial ganglionic eminence GABAergic interneurons cells (NRTX-1001) in the hippocampus [147], and clinical observations are consistent with preclinical results reported in a mouse model of mesial TLE [93]. Multiple phase 1/2 studies are currently examining ETX101, an AAV serotype 9 (AAV9) gene therapy designed to express voltage-gated sodium channels (Na_V_1.1) in GABAergic inhibitory interneurons [64] in children with *SCN1A*-positive Dravet syndrome [148,149,150]. These studies rely on preclinical efficacy (disease-modifying) and safety reported in *Scn1a*^+/−^ knockout mice and nonhuman primates, respectively [64]. Additionally, a phase 1/2 study examining the safety, tolerability, and efficacy of AMT-260, an AAV9 vector that reduces expression of GluK2 and GluK2-containing kainate receptors, is being conducted in adults with unilateral refractory mesial TLE [151]. In phase 1/2a trials of zorevunersen (STK-001), an antisense oligonucleotide that increases expression of Na_V_1.1 channels [152], median convulsive seizure frequency was reduced at 3 and 6 months after the last dose compared with baseline in children with *SCN1A*-positive Dravet syndrome [153,154]. The efficacy, safety, and tolerability of zorevunersen will be further examined in a phase 3, multicenter, randomized, double-blind, sham-controlled clinical trial [155].

## 5. Conclusions

Exciting steps are being taken to study disease modification in epilepsy. Major problems that remain are the tenuous link between animal models and human epilepsies and the huge variety of types of seizures, epilepsies, and variations in the pathophysiology of human epilepsy. For example, the methods typically used to induce SE in animals differ from the common causes of SE in humans (e.g., stroke and brain trauma) [156]. Some preclinical methods in animals that induce seizures and epilepsy in relatively rapid fashion are synthetically induced (e.g., chemical or electrical stimuli), and the methods themselves might influence study outcomes apart from epileptogenesis. Homogeneity in preclinical methods, including animal models and strains, is best for reducing variability and detecting statistically significant differences but may affect generalizability to human epilepsy. Moreover, human brains differ substantially from animal brains and may respond differently to identical insults. Commonalities in underlying neural mechanisms, however, provide hope that results from some animal models will transfer to humans. It is hoped that better characterization of small-molecule therapies and newer therapeutic techniques will provide insights that allow for modification of disease course and improvement in patient lives.

## Figures and Tables

**Table 1:** Summary of Published Preclinical Results.

Therapy			Model	AE or DM	Outcomes			Author	Year
Class	Name	Type			SRS	Behavioral Comorbidities	Pathology		
**Genetic Epilepsy**									
Antidepressant	Duloxetine	Small molecule	Absence epilepsy	DM	Improved	No change	NT	Citraro [60]	2015
Anti-inflammatory	Fingolimod	Small molecule	Absence epilepsy	DM	Improved	Improved	NT	Leo [61]	2017
Anti-inflammatory	Tocilizumab	Antibody	Absence epilepsy	DM	Improved	Improved	NT	Leo [62]	2020
ASM	Perampanel	Small molecule	Absence epilepsy	DM	Improved	Improved	NT	Citraro [58]	2017
ASM	Ethosuximide	Small molecule	Absence epilepsy	DM	Improved	Improved	NT	Dezsi [57]	2013
Neuronal excitability	AAV9.hCLN2	Gene therapy	CLN2 disease	DM	Improved	NT	Improved	Takahashi [63]	2023
Neuronal excitability	AAV9-RE^GABA^-eTF^SCN^	Gene therapy	Dravet syndrome	DM	Improved	NT	NT	Tanenhaus [64]	2022
**Acquired Epilepsy (TLE)**									
Anti-inflammatory	Anakinra	Small molecule	Post-SE	AE/DM	Improved	Improved	NT	Dyomina [65]	2020
Anti-inflammatory	Anti-HMGB1 monoclonal antibody (anti-HMGB1 mAB)	Antibody	Post-SE	AE/DM	Improved	NT	NT	Zhao [66]	2017
Anti-inflammatory	Everolimus	Small molecule	Post-SE	No effect	No change	NT	NT	Barker-Haliski [54]	2021
Anti-inflammatory	Fingolimod	Small molecule	Post-SE	AE	Improved	NT	NT	Pitsch [67]	2019
Anti-inflammatory	Intravenous immunoglobulin	Small molecule	Post-SE	AE/DM	Improved	NT	Improved	Chen [68]	2017
Anti-inflammatory	JNJ-47965567	Small molecule	Post-SE	DM?	Improved	NT	Improved	Jimenez-Pacheco [69]	2016
Anti-inflammatory	MicroRNA-146a	Micro-RNA	Post-SE	DM	Improved	NT	NT	Iori [52]	2017
Anti-inflammatory	Minocycline	Small molecule	Viral infection	DM	NT	Improved	NT	Barker-Haliski [70]	2016
Anti-inflammatory	Minocycline	Small molecule	Post-SE	AE/DM	Improved	NT	Improved	Wang [71]	2015
Anti-inflammatory	Parecoxib	Small molecule	Post-SE	No effect	No change	No change	NT	Polascheck [72]	2010
Anti-inflammatory	PQR620, PQR530	Small molecule	Post-SE	DM?	No change	Improved	NT	Gericke [73]	2020
Anti-inflammatory	Saracatinib (AZD0530)	Small molecule	Post-SE	AE/DM	Improved	NT	Improved	Sharma [74]	2021
Antioxidant	1400W	Small molecule	Post-SE	AE/DM	Improved	NT	NT	Puttachary [75]	2016
Antioxidant	Curcumin	Small molecule	Post-SE	DM	Improved	Improved	NT	Jiang [76]	2015
Antioxidant	Dimethyl fumarate	Small molecule	Post-SE	AE/DM	Improved	Improved	Improved	Sandouka [77]	2023
Antioxidant	Losartan	Small molecule	Post-TBI	AE	Improved	NT	NT	Bar-Klein [78]	2014
Antioxidant	Losartan	Small molecule	Post-SE	AE/DM	Improved	Improved	NT	Tchekalarova [79]	2014
Antioxidant	Miconazole	Small molecule	Post-SE	DM	Improved	NT	Improved	Gong [80]	2022
Antioxidant	RTA408	Small molecule	Post-SE	AE	Improved	NT	NT	Shekh-Ahmad [81]	2018
ASM	Cannabidiol	Small molecule	Kindle	DM	Improved	NT	NT	Reddy [50]	2023
ASM	Carbamazepine	Small molecule	Post-SE	No effect	No change	NT	NT	Iori [52]	2017
ASM	Lacosamide	Small molecule	Post-SE	No effect	No change	NT	Improved	Licko [56]	2013
ASM	Lacosamide	Small molecule	Post-SE	AE?	?	NT	NT	Wasterlain [55]	2011
ASM	Lamotrigine	Small molecule	Post-SE	AE	Improved	NT	Improved	Wang [51]	2019
ASM	Levetiracetam	Small molecule	Post-SE	No effect	No change	No change	No change	Brandt [82]	2007
ASM	Phenobarbital	Small molecule	Post-SE	No effect	No change	NT	NT	Barker-Haliski [54]	2021
ASM	Valproate	Small molecule	Post-SE	No effect	No change	NT	Improved	Langer [53]	2011
Diet/microbiome	Sodium selenate	Small molecule	Post-SE	AE	Improved	Improved	NT	Casilla-Espinosa [83]	2023
Kinase inhibitor	5-iodotubercidin	Small molecule	Post-SE	AE	Improved	NT	Improved	Sandau [84]	2019
Neuronal Excitability	Antagomirs	ASO	Post-SE	AE	Improved	NT	NT	Reschke [85]	2017
Neuronal excitability	Antagomirs	ASO	Post-SE	AE	Improved	NT	NT	Reschke [86]	2021
Neuronal excitability	Ascl1 and Dlx2 reprogram reactive glia	Stem cell	Post-SE	DM	Improved	NT	NT	Lentini [87]	2021
Neuronal excitability	Biperiden	Small molecule	Post-SE	AE	Improved	No change	NT	Bittencourt [88]	2017
Neuronal excitability	Bumetanide	Small molecule	Post-SE	No effect	No change	NT	NT	Brandt [89]	2010
Neuronal excitability	Deep brain stimulation	Brain stimulation	Post-SE	DM	Improved	NT	NT	Costard [90]	2019
Neuronal excitability	Embryonic medial ganglionic eminence progenitor cells	Stem cell	Post-SE	DM	Improved	Improved	NT	Casalia [91]	2017
Neuronal excitability	Hippocampal grafts	Stem cell	Post-SE	DM	Improved	NT	Improved	Henderson [92]	2014
Neuronal excitability	Human embryonic stem cell line	Stem cell	Post-SE	DM	Improved	NT	Improved	Bershteyn [93]	2023
Neuronal excitability	NS1209	Small molecule	Post-SE	AE	No change	NT	Improved	Langer [53]	2011
Neuronal excitability	RHC80267	Small molecule	Post-SE	AE	Improved	Improved	NT	Ma [94]	2014
Neuronal excitability	Scopolamine	Small molecule	Post-SE	AE	Improved	Improved	NT	Meller [95]	2021
Neuronal excitability	Scopolamine	Small molecule	Post-SE	AE	Improved	NT	NT	Pereira [96]	2005
Neuronal excitability	Trilostane	Small molecule	Post-SE	DM	Improved	NT	NT	Gol [97]	2024
Neuronal excitability	Z944	Small molecule	Post-SE	DM	Improved	NT	NT	Casilla-Espinosa [98]	2019
Neurotrophic	AAV-pDyn	Gene therapy	Post-SE	DM	Improved	Improved	NT	Agostinho [99]	2019
Neurotrophic	Cintrofin	Small molecule	Post-SE	DM?	No change	Improved	NT	Russmann [100]	2013
Neurotrophic	Glial cell line-derived neurotrophic factor	Small molecule	Post-SE	DM	Improved	Improved	NT	Paolone [101]	2019

AE, antiepileptogenic; ASM, antiseizure medication; ASO, antisense oligonucleotide; DM, disease-modifying; mAB, monoclonal antibodies; NT, not tested; SE, status epilepticus; SRS, spontaneous recurring seizures; TBI, traumatic brain injury; TLE, temporal lobe epilepsy; ?, possibly supportive. Antiepileptogenic therapeutic strategy prevented, delayed, or reduced the severity of the onset of epilepsy (SRS). Disease-modifying therapeutic strategy modified disease course when administered to animals with established SRS. Evidence of disease modification includes any positive change in SRS, behavior, and/or pathology. A change in any one of these domains qualifies as disease modification.

**Table 2:** Summary of Published Clinical Results.

Therapy		Epilepsy	Key Findings	Author	Year	Study Type
Class	Name					
ASM	Cenobamate	Focal	● Median percent change from baseline in seizure frequency during 18-week treatment period was 55% with cenobamate vs. 24% with placebo	Krauss [120]	2020	RCT
ASM	Cenobamate	Focal	● Seizure freedom during consecutive 6-month intervals increased from 25% (months 3–9) to 44% (months 27–33) with cenobamate	Sperling [121]	2021	OL
ASM	Cannabidiol	DEE (LGS)	● Median percent reduction in total seizures increased from 48% (weeks 1–12) to 65% (weeks 145–156) with cannabidiol oral solution	Patel [122]	2021	OLE
ASM	Cannabidiol	DEE (DS)	● Median percent reduction in total seizures increased from 49% (weeks 1–12) to 78% (weeks 145–156) with cannabidiol oral solution	Scheffer [123]	2021	OLE
ASM	Vigabatrin	DEE (TSC)	● 65% of children with TSC treated within 1 week of seizure onset achieved seizure freedom compared with 24% who had delayed treatment (3 weeks or later after seizure onset)	Cusmai [124]	2011	Retrospective, Cohort
ASM	Vigabatrin	DEE (TSC)	● Children with TSC and early treatment (within 1 week of epileptiform discharges) had lower rates of intellectual disability (14% vs. 48%), polytherapy (21% vs. 55%), and DRE (7% vs. 42%) than those with standard treatment (within 1 week of seizure onset)	Jozwiak [125]	2011	Prospective, Cohort
ASM	Vigabatrin	DEE (TSC)	● Children with TSC treated before detection of epileptiform activity had lower odds (OR, 95% CI) of clinical seizures (0.21, 0.04–0.9), infantile spasms (0, 0–0.33), and DRE (0.23, 0.06–0.83) compared with treatment after electrographic or clinical seizures ● No treatment differences in cognitive scores or incidence of neurodevelopmental delay	Kotulska [126]	2021	RCT
ASM	Vigabatrin	DEE (TSC)	● Children treated with vigabatrin at first detection of epileptiform EEG had similar rates of seizures or DRE compared with placebo ● Vigabatrin delayed the onset and decreased the prevalence of infantile spasms ● No treatment differences in cognitive or behavioral outcomes	Bebin [127]	2023	RCT
ISM	Diazepam nasal spray	Focal or generalized epilepsy	● Mean interval between seizure clusters (SEIVAL) increased from 13.9 days (Period 1, days 1–90) to 26.8 days (Period 4, days 271–360)	Misra [128]	2022	OL
Anti-inflammatory	Everolimus	DEE (TSC)	● Proportion of patients with ≥50% reduction in seizure frequency was greater in low-dose (28.2%) and high-dose (40.0%) everolimus groups than in placebo (15.1%) ● Median percent change in seizure frequency was 14.9%, 29.3%, and 39.6% in placebo, low-dose, and high-dose everolimus groups, resp.	French [129]	2016	RCT
Anti-inflammatory	Everolimus	DEE (TSC)	● White matter changes associated with TSC were modified with everolimus	Peters [130]	2019	OL/OLE
Anti-inflammatory	Dexamethasone	DRE	● IEA burden was reduced after dexamethasone treatment compared with baseline ● Sleep physiology and QOL improved	Schiller [131]	2024	Retrospective, cohort

ASM, antiseizure medication; CI, confidence interval; DEE, developmental epileptic encephalopathy; DRE, drug-resistant epilepsy; DS, Dravet syndrome; EEG, electroencephalogram; IEA, interictal epileptic activity; ISM, immediate-use seizure medication; LGS, Lennox–Gastaut syndrome; OL, open-label; OLE, open-label extension; OR, odds ratio; QOL, quality of life; RCT, randomized controlled trial; SEIVAL, seizure interval; TSC, tuberous sclerosis complex.

## Data Availability

Not applicable.
